# Supplementary figures and images for: Development and validation of the Physical Capacity Score (PiC) to overcome the lack of correlation among traditional physical tests in detecting age-related decline
Source: PLoS One. 2026 Feb 19;21(2):e0343122. doi: 10.1371/journal.pone.0343122 (PMC12919782; doi:10.1371/journal.pone.0343122)

**S1 Appendix. Bland-Altman plots for each test and subject.**


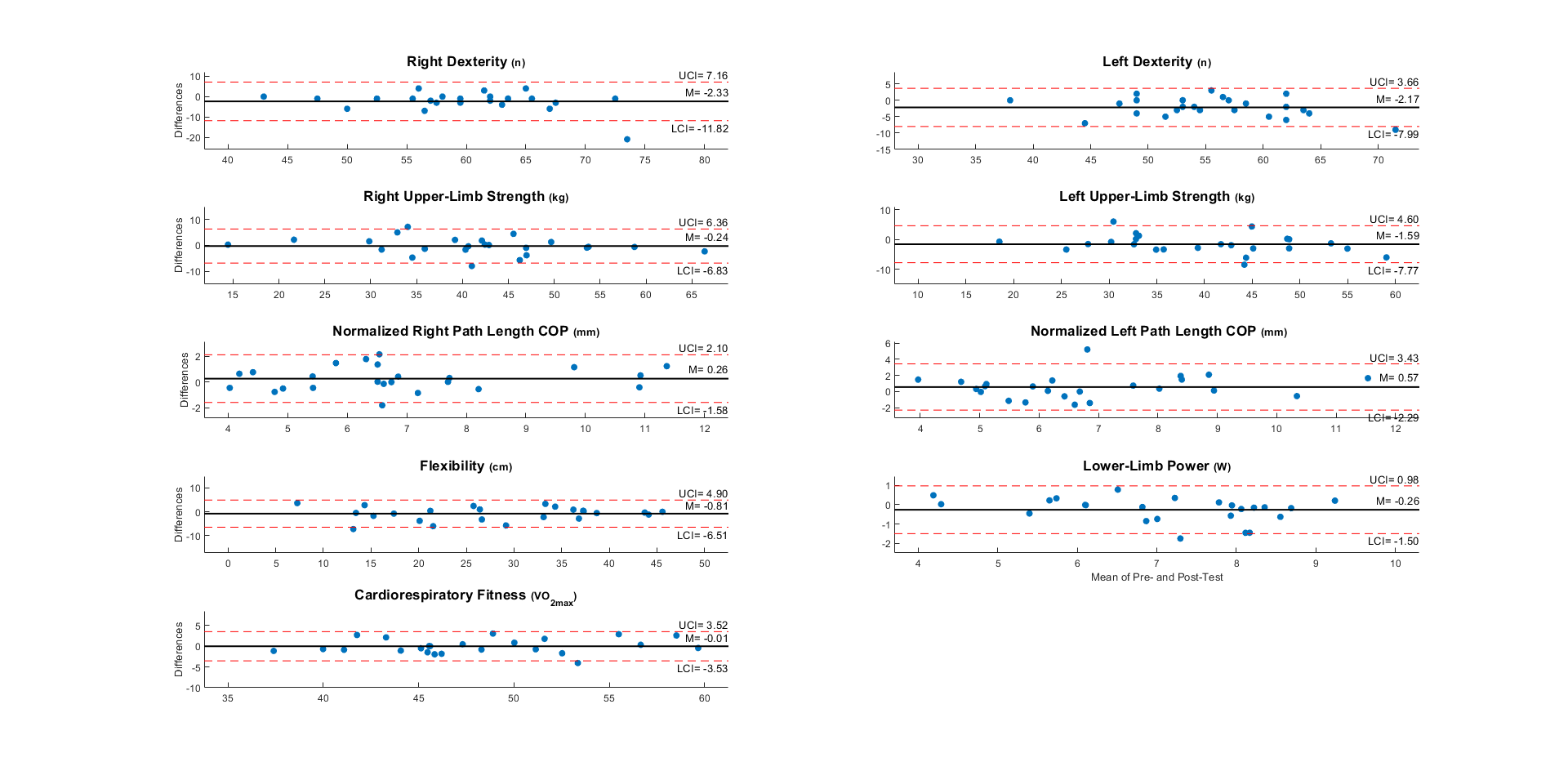

Supplement: S1 Appendix — (DOCX) [file pone.0343122.s001.docx]
